# Use telehealth as needed: telehealth substitutes in-person primary care and associates with the changes in unplanned events and follow-up visits

**DOI:** 10.1186/s12913-023-09445-0

**Published:** 2023-05-03

**Authors:** Ying (Jessica) Cao, Dandi Chen, Maureen Smith

**Affiliations:** 1 Department of Population Health Sciences, University of Wisconsin-Madison, 610 Walnut St., 760B WARF Office Building, Madison, WI 53726 USA; 2 Department of Family Medicine and Community Health, University of Wisconsin-Madison, 610 N Whitney Way, Ste 200, Madison, WI 53705 USA; 3 Health Innovation Program, University of Wisconsin-Madison, 800 University Bay, Madison, WI 53726 USA

**Keywords:** Telehealth, Emergency department visits, Hospitalization, Follow-up visits

## Abstract

**Background:**

Telehealth rapidly expanded since the outbreak of the COVID-19 pandemic. This study aims to understand how telehealth can substitute in-person services by 1) estimating the changes in non-COVID emergency department (ED) visits, hospitalizations, and care costs among US Medicare beneficiaries by visit modality (telehealth vs. in-person) during the COVID-19 pandemic relative to the previous year; 2) comparing the follow-up time and patterns between telehealth and in-person care.

**Methods:**

A retrospective and longitudinal study design using US Medicare patients 65 years or older from an Accountable Care Organization (ACO). The study period was April-December 2020, and the baseline period was March 2019 – February 2020. The sample included 16,222 patients, 338,872 patient-month records and 134,375 outpatient encounters. Patients were categorized as non-users, telehealth only, in-person care only and users of both types. Outcomes included the number of unplanned events and costs per month at the patient level; number of days until the next visit and whether the next visit happened within 3-, 7-, 14- and 30-days at the encounter level. All analyses were adjusted for patient characteristics and seasonal trends.

**Results:**

Beneficiaries who used only telehealth or in-person care had comparable baseline health conditions but were healthier than those who used both types of services. During the study period, the telehealth only group had significantly fewer ED visits/hospitalizations and lower Medicare payments than the baseline (ED 13.2, 95% CI [11.6, 14.7] vs. 24.6 per 1,000 patients per month and hospitalization 8.1 [6.7, 9.4] vs. 12.7); the in-person only group had significantly fewer ED visits (21.9 [20.3, 23.5] vs. 26.1) and lower Medicare payments, but not hospitalizations; the both-types group had significantly more hospitalizations (23.0 [21.4, 24.6] vs. 17.8). Telehealth was not significantly different from in-person encounters in number of days until the next visit (33.4 vs. 31.2 days) or the probabilities of 3- and 7-day follow-up visits (9.2 vs. 9.3% and 21.8 vs.23.5%).

**Conclusions:**

Patients and providers treated telehealth and in-person visits as substitutes and used either depending on medical needs and availability. Telehealth did not lead to sooner or more follow-up visits than in-person services.

**Supplementary Information:**

The online version contains supplementary material available at 10.1186/s12913-023-09445-0.

## Background

Telehealth has rapidly expandedworldwide as an alternative delivery mode to in-person care since the outbreak of the COVID-19 pandemic [1–4]. Over the past two years and especially at the beginning of the pandemic, telehealth relieved the burden to the whole healthcare system by sparing more labor and physical resources taking care of the COVID cases [5, 6]. It also protected patients and care providers from the risk of infection while maintaining timely access to necessary care services [7–10]. Existing research on telehealth since then have shown the changing volume over time [11–13], share and concentration across care service areas [2, 14], as well as utilization by patient subgroup [15–17] and among those with special needs [18–21]. However, there is limited evidence about the effects of telehealth on long-term healthcare costs and patient outcomes [22, 23]. Also limited is the knowledge on the relative timing (e.g., one type of service happens before or after the other) and the subsequent utilization patterns (e.g., follow-up rates and visit types) between telehealth and in-person options, which would also influence the direction and magnitude of telehealth effects on care costs and outcomes [24–27].

There have been mixed opinions on the effects of telehealth. On the one hand, people believe that telehealth provides timely care access and in a relatively cost-friendly way. As a result, it could reduce emergency department (ED) visits and hospitalizations that would otherwise be avoidable and ultimately save healthcare costs by reducing unplanned events [28–31]. Conversely, some argue that telehealth is not an adequate substitute for in-person service, and will either need in-person follow-up visits to supplement the service or cause even more delayed or missed care, resulting in higher care costs, worse care outcomes, or both [24, 25, 32]. Uncertainties to these questions impede policy makers and private entities such as insurers, clinicians and/or care organizations to make permanent decisions on telehealth when the public health emergency is coming to an end [33–38].

Telehealth also imposes unique challenges and opportunities for care delivery among the aging population worldwide. Take the US as an example. The Centers for Medicare & Medicaid Services (CMS) has allowed telehealth to be reimbursed for the traditional Medicare fee-for-service beneficiaries since the COVID-19 pandemic started in March 2020 [8, 16, 35, 38]. CMS is a US federal agency that administers the Medicare program (i.e. health insurance for the aging population and those with disabilities) and works in partnership with state governments to administer Medicaid (i.e. health insurance for the low-income population) [39]. Since then, the volume of telehealth delivery increased more than ten-fold within the first month of this policy change [35, 38]. Care systems and clinics also actively incorporated telehealth in primary care settings and chronic condition management programs [19, 20, 40]. Older and more vulnerable patients viewed telehealth as a more attractive option than risking interaction with others, especially in the waiting areas. However, disparity issues and barriers to access to telehealth also existed among historically underserved groups [41, 42]. During the COVID-19 pandemic, telehealth utilization was consistently lower among racial/ethical minorities, the low-income population, the advanced aged and those from remote rural areas [16, 43]. The CMS reimbursement policy change offers a unique opportunity to evaluate the utilization patterns and the effectiveness of telehealth relative to in-person services among the aging population when compared to the pre-policy settings [8, 16].

The objective of this study is to assess the extent to which telehealth can substitute in-person visits in primary care settings by 1) examining the association of changes in unplanned events (e.g., ED visits and hospitalizations) with visit modality (telehealth vs. in-person) during the COVID-19 pandemic relative to the previous years; and 2) comparing the visit time and pattern between telehealth and in-person care. The study first used longitudinal patient-level care utilization data to compare the occurrence and number of unplanned events and the healthcare costs by patients’ telehealth and in-person utilization status (none, either or both). This study also used encounter-level data to estimate the time duration until the next visit and the likelihood of follow-up visits for telehealth and in-person delivery modes. It is hypothesized that patient choose to use telehealth upon available and medical needs, and telehealth is comparable to in-person services in follow-up visit patterns. Results from the study fill in the knowledge gap by providing evidence of using telehealth as a substitute for in-person services. The evidence is critical for stakeholders to make long-term decisions on telehealth delivery such as regulations, reimbursement, as well as inclusion in the primary care settings and/or chronic care management programs. Experiences learnt from the US healthcare system on telehealth delivery and healthcare quality improvement will also benefit other countries across the world.

## Methods

### Data sources and the study sample

This study adopted a retrospective design and used electronic health records and claims data for Medicare fee-for-service beneficiaries served by an Accountable Care Organization [44] (ACO) in the Midwest of the United States. The study period was defined as April to December 2020, and the baseline period was from March 2019 to February 2020. March 2020 was excluded since the COVID-19 pandemic started in March and made the month less comparable to other months in the study time window. Beneficiaries who were 65 years or older and with a primary care physician (PCP) in the ACO provider list at the beginning of the study period were included in the analysis. Telehealth was coded from date of patient receipt of primary care services by audio or video telecommunication using standard billing (CPT/HCPCS) codes [4, 17, 26, 45–47].

Two types of the data were used for analysis. First, the non-COVID related care utilization data was collapsed into patient-month level and was used as longitudinal data throughout the observational period: 12-month baseline and 9-month study period. Second, the outpatient encounter-level data was organized by beneficiary and encounter date. One encounter represents one primary care visit (in-person or telehealth). A patient could have none, one or more than one in-person or telehealth encounter in any month. This encounter-level data was used to compare telehealth and in-person visit utilization and follow-up patterns. This study is exempted by the Institutional Review Board in the University of Wisconsin-Madison. Reporting of the study follows the STROBE checklist for observational studies.

### Outcomes

For the patient level analysis, outcomes are the occurrence of unplanned events (ED visits and hospitalizations) and the total unplanned Medicare payment ($) in each month. To differentiate care utilization at the intensive and extensive margin, the occurrence of unplanned events was measured as the number of ED visits or hospitalizations as well as whether there were at least one ED visits or hospitalizations in a month. For the encounter-level analysis, outcomes are the number of days until the next encounter and further, whether the next encounter happened within the 3-, 7-, 14- and 30-day frame.

### Major explanatory variables

In the patient-level analysis, the two major explanatory variables were a binary indicator of the study period relative to baseline, and a categorical variable to differentiate patients by their outpatient utilization throughout the entire study period: whether a patient had no visit, telehealth visits only, in-person visits only, and visits of both types—in-person and telehealth. Different from other existing studies which used telehealth utilization status as the sole indicator [24–27], this study considered the co-existence of in-person visits and categorized beneficiaries into four mutually exclusive subgroups. We hypothesized that patients who used only telehealth and those who used both types of services were different and should not be grouped together when compared with the patients who did not use telehealth (the in-person only group). For the encounter-level analysis, the two major explanatory variables were a binary indicator of the study period during the pandemic relative to the pre-pandemic baseline period, and an indicator of the encounter type: telehealth relative to in-person visits.

### Control variables

For both patient- and encounter-level analysis, control variables included sociodemographic variables, health conditions, care utilization at the baseline as well as month fixed effects to control for seasonal trends. Inclusion of these variables are based on data availability and the Andersen's Model of Health Care Utilization [48] including predisposing factors such as a beneficiary’s age group (65–74 years old, 75–85, and 86 +), sex (0/1), race/ethnicity (non-Hispanic White or minorities); socioeconomic factors such as Medicaid enrollment (0/1), disability entitlement (0/1) and the geographical residence category (urban, suburban, large town, small town/isolated rural); and need characteristics such as the hierarchical condition categories (HCC) score, an indicator of having three or more chronic conditions (0/1), the total number of unplanned event counts (ED visits and hospitalizations) and payments at the baseline period.

### Statistical approaches

For the patient-level analysis, logistic regression models were used to predict the probability of having at least one ED visit or hospitalization per patient per month. Negative binomial models were used to predict the number of ED visits or hospitalizations. Ordinary least square models were used to predict the average logarithm of unplanned payments. All models were controlled for patient characteristics at baseline and the month fixed effects. The predicted probabilities, number of unplanned events and dollar value of payments were reported.

For the encounter-level analysis, we first plotted the distributions of the number of days until the next visit for all encounters by time (pre-pandemic period vs. during) and the encounter type (telehealth vs. in-person visits). It was hypothesized that if a patient used either telehealth or in-person encounter type as a substitute for the other, the distributions of time until the next visit should be comparable between the two encounter types. In addition, we used Poisson regression models to estimate the time duration until the next visit and used logistic regression models to estimate the probabilities that the next visit happened within 3, 7, 14 and 30 days since the last visit. These models were adjusted for the pandemic time indicator, encounter type and month fixed effects. The incidence rate ratios (IRR)/odds ratios (OR) from the regressions, the predicted number of days until the next visit and the probabilities of 3-, 7-, 14-, and 30-day follow-up were reported.

Including patient-level characteristics at the baseline period and the month fixed effects in all analysis helped to absorb the potential confounding bias at the patient-level or due to seasonal trends. Robust standard error was also used to refine estimates. Records with missing data, which accounted for less than 1% of the study sample, were dropped from the analysis.

## Results

### Sample characteristics

The study sample included 338,872 patient-month records and 134,375 outpatient encounters during the observational period (March 2019 to December 2020) for a total of 16,222 beneficiaries. The study sample had a mean age of 76 (standard deviation SD 6.5) years old, 61% female, 96% non-minority White, 6.5% with Medicare & Medicaid dual coverages, 4.3% with disability entitlements, 70% from urban areas, 19% suburban and 11% from large, small town or remote rural areas (Table 1).

**Table 1:** Baseline Characteristics of Beneficiaries by Outpatient Utilization Category

	Beneficiaries (*N* = 16,222)			
**Baseline Characteristics**	No Visit in Study Period (*N* = 2,976)	Only Telehealth Visits (*N* = 2,929)	Only In-person Visits (*N* = 4,853)	Both Telehealth and In-person (*N* = 5,464)
***Sociodemographic***				
Mean Age (SD)	74.53 (6.56)	74.85 (6.53)	74.81 (6.25)	75.86 (6.61)
Age 65–74, %	60.52	57.60	57.04	49.96
Age 75–85	28.93	32.02	34.19	38.45
Age 86 +	10.55	10.38	8.78	11.58
Female	59.91	61.66	58.46	62.06
Race, %				
Non-Hispanic White	95.67	96.07	96.25	96.32
Other	4.33	3.93	3.75	3.68
Medicaid Insurance Ever, %	6.05	5.57	4.62	6.57
Disability Entitlement, %	3.76	4.47	3.75	4.92
Rural/Urban, %				
Urban	70.80	74.05	64.87	70.31
Suburban	19.76	16.01	21.80	18.28
Large Town	8.17	8.54	11.79	10.21
Small Town/Isolated Rural	1.28	1.40	1.55	1.17
***Health Conditions***				
Mean HCC Score (SD)	0.93 (0.99)	1.02 (1.04)	0.972 (0.899)	1.43 (1.26)
3 or More Chronic Conditions %	31.32	47.90	40.96	64.00

*SD* Standard deviation. Entries are percentages (0–100) unless otherwise noted

During the 9-month study period (April to December 2020), 18.3% (*N* = 2,976 out of 16,222) of the beneficiaries had no outpatient visit, 18.1% (*N* = 2,929) had only telehealth visits, 29.9% (*N* = 4,853) had only in-person visits, and 33.7% (*N* = 5,464) had both types. Comparing across the four outpatient encounter utilization categories by baseline characteristics, those beneficiaries who used both telehealth and in-person visits were older (76 vs. 75 yrs.), had a higher average HCC score (1.43 vs. 0.93–1.02) and were more likely to have 3 or more chronic conditions (64% vs. 31–48%) than those from the other three categories. Comparing between the telehealth only and in-person only groups, the in-person only group had more male beneficiaries (42% vs. 38%), more between 75–85 years old (34% vs. 32%), less 86 years or older (8.8% vs. 10.4%) or from urban areas (65% vs. 74%). The average HCC scores were comparable but the probability of having 3 or more chronic conditions were lower in the in-person only group than the telehealth only group (41% vs. 48%). These results provided initial evidence that less healthy beneficiaries or those with more clinical needs used both telehealth and in-person visits, while those healthier ones chose either type to use by their preferences (Table 1).

### Predicted probability and number of ED visits

After risk adjustment, the average predicted probability of having at least one ED visit per month after adjusting all patient characteristics during the baseline period (March 2019 – February 2020) were comparable across the three healthier groups (2.02%, 2.21% and 2.32% for the no-visit, telehealth-only and in-person only group). The group that used both types of visits had a significantly higher baseline probability at 3.5% (95% CI [3.4%, 3.6%]). In comparison, during the study period, the predicted probability for no visit, telehealth-only and the in-person only groups significantly decreased relative to their baseline levels (0.77% CI [0.7%. 0.9%], 1.23% [1.1%, 1.4%], and 2.01% [1.9%, 2.2%]). But for the group that used both types of visits, the predicted probability of have at least one ED visit per month did not significantly change from the baseline (3.52% [3.4%, 3.7%]) (Table 2 & Supplementary Table 1).

**Table 2:** Risk-Adjusted Occurrence of Unplanned Events and Medicare Payments per Month

	Patient-Month Records (*N* = 338,872)			
	No Visits (*N* = 61,548)	Telehealth Only (*N* = 61,235)	In-person Only (*N* = 101,695)	Both Types (*N* = 114,394)
Prob (ED = 1) per month (%)				
Baseline	2.02 (1.87, 2.17)	2.21 (2.06, 2.37)	2.32 (2.20, 2.45)	3.50 (3.37, 3.64)
Study Period	0.77 (0.65, 0.88)	1.23 (1.10, 1.37)	2.01 (1.87, 2.15)	3.52 (3.36, 3.68)
Difference	-1.25 (-1.52, -0.99) ^a^	-0.98 (-1.27, -0.69) ^a^	-0.31 (-0.58, -0.05) ^a^	0.02 (-0.28, 0.3)
# of ED per 1,000 patients per month				
Baseline	22.5 (20.8, 24.3)	24.6 (22.8, 26.4)	26.1 (24.5, 27.6)	40.1 (38.4, 41.7)
Study Period	8.2 (6.9, 9.5)	13.2 (11.6, 14.7)	21.9 (20.3, 23.5)	41.8 (39.6, 43.9)
Difference	-14.3 (-17.4, -11.3) ^a^	-11.4 (-14.8, -8.1) ^a^	-4.2 (-7.3, -1) ^a^	1.7 (-2.1, 5.5)
Prob (Hospitalization = 1) per month (%)				
Baseline	0.98 (0.88, 1.10)	1.14 (1.03, 1.25)	1.13 (1.04, 1.22)	1.55 (1.46, 1.63)
Study Period	0.46 (0.37, 0.55)	0.69 (0.58, 0.79)	1.03 (0.92, 1.13)	1.87 (1.78, 1.99)
Difference	-0.52 (-0.73, -0.33) ^b^	-0.45 (-0.67, -0.24) ^a^	-0.1 (-0.3, 0.09)	0.32 (0.15, 0.53) ^b^
# of Hospitalization per 1,000 patients per month				
Baseline	11.0 (9.7, 12.3)	12.7 (11.4, 14.0)	12.9 (11.7, 14.1)	17.8 (16.8, 18.9)
Study Period	5.5 (4.3, 6.7)	8.1 (6.7, 9.4)	12.3 (10.9, 13.7)	23.0 (21.4, 24.6)
Difference	-5.5 (-8, -3) ^a^	-4.6 (-7.3, -2) ^a^	-0.6 (-3.2, 2)	5.2 (2.5, 7.8) ^a^
$ Unplanned payment per patient per month				
Baseline	4772 (4283, 5260)	7326 (6590, 8062)	7184 (6467, 7901)	13,449 (12,167, 14,731)
Study Period	1896 (1700, 2092)	3778 (3393, 4163)	4838 (4350, 5325)	12,588 (11,365, 13,790)
Difference	-2876 (-3560, -2191) ^a^	-3548 (-4669, -2427) ^a^	-2346 (-3551, -1142) ^a^	-861 (-3366, 1623)

95% confidence interval in parentheses. ^a^99%, ^b^95% significant level

Entries are predicted occurrence of unplanned events (Emergency Department (ED) visits and hospitalization) and Medicare payment after risk adjustment of patient characteristics and month fixed effects. Occurrence of ED visits (or hospitalization) were measured by the average probability of having at least 1 ED visits (or hospitalizations) per patient per month and the average number of ED visits (or hospitalizations) per 1,000 patients per month. Medicare payment was measured by the average dollar amount per patient per month. Patient characteristics included age category (65–74 yrs., 75–85 yrs. or 86 + yrs.), sex, race/ethnicity (non-Hispanic White or Others), Medicaid coverage (0/1), disability entitlement (0/1), rural/urban residence (urban, suburban, large town, or small town/isolated rural), Hierarchical Condition Category (HCC) score and having 3 or more chronic conditions (0/1)

The predicted number of ED visits before and during the pandemic across the four care utilization groups followed similar patterns. In the baseline period before the pandemic, the estimated average number of ED visits per 1,000 patients per month were comparable at 22.5, 24.6 and 26.1 (±1.8) visits for the no-visit, telehealth-only and in-person only groups and was significantly higher at 40.1 (95% CI [38.4, 41.7]) visits for the both-types group. During the pandemic, the first three groups significantly reduced the number of ED visits (8.2, 13.2 and 21.9, ±1.6) while the fourth group did not show significant change from before (41.8 CI [39.6, 43.9]) (Table 2).

Comparing across the no-visit, telehealth-only and in-person only groups before and during the pandemic, the three comparable groups in ED visits during the baseline became significantly different from each other during the pandemic. The in-person group experienced the least reduction in ED visits, yielding the highest probability and number of ED visits among the three, while the no-visit group was the opposite. Comparing between telehealth-only and in-person only groups, the two groups had comparable baseline health conditions and ED visits, but the in-person only group had significantly higher predicted probability and number of ED visits than the telehealth-only group during the pandemic. Overall, these results suggested that patients used either telehealth or in-person visits or both depending on their clinical needs and availability and treated the two types of care delivery modes as a substitute to each other.

### Predicted probabilities and number of hospitalizations

The changing patterns of the predicted probability and number of hospitalizations across the four care utilization groups were similar to that of the ED visits in the baseline period but were different during the pandemic. In the baseline period, the number of hospitalizations for the no-visit, telehealth-only and in-person only groups were comparable among each other (at 11.0, 12,7 and 12.9±1.6 hospitalizations per 1,000 patients per month respectively) and were significantly lower than the both-types group (17.8 CI [16.8, 18.9]). During the pandemic, the no-visit and telehealth-only groups had significantly fewer hospitalizations relative to the pre-pandemic period (5.5 and 8.1±1.6). The in-person only group showed no significant changes (12.3 CI [10.9, 13.7]) and the both-types groups had a significantly higher number of hospitalizations than before (23.0 CI [21.4, 24.6]) (Table 2).

### Predicted payment for unplanned events

In the baseline period, the average monthly payment per person for the telehealth and in-person groups were comparable between each other ($7,326 and $7,184) but were significantly higher than the no-visit group ($4,772) and lower than the both-types group ($13,449). During the pandemic, the no-visit, telehealth only, and in-person only groups had lower payments than pre-pandemic levels ($1,896, $3,778, and $4,838 respectively), but the both-types group had no significant changes from before ($12,588). Further, the estimated payments for the telehealth only and in-person only groups became significantly different from each other during the pandemic (mean difference $1,060, CI [$957, $1,162]) (Table 2).

### Follow-up patterns for telehealth and in-person visits

**Fig. 1:**
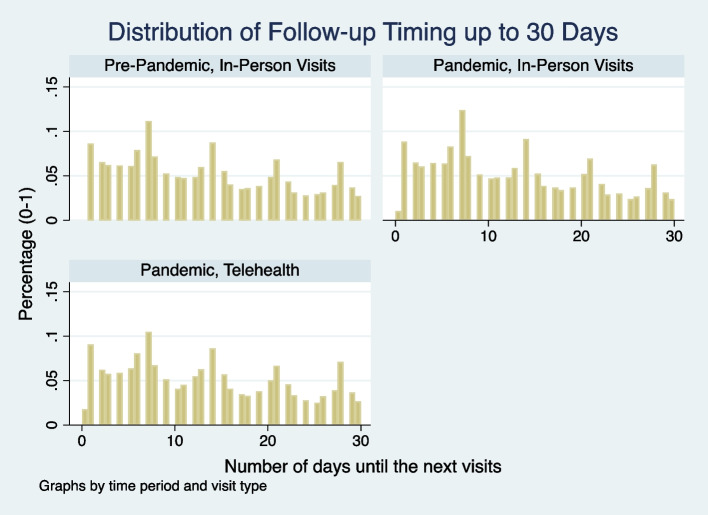
Distribution of Number of Days until the Next Visit by Encounter Type Before and During the Pandemic. Note: Sample included in-person and telehealth encounters (/visits) for the pandemic cohort (65 yrs. or older with a UW PCP) pre- and during pandemic without March 2020. The distributions of number of days until the next visits were comparable both before and during the pandemic and between telehealth and in-person visits during the pandemic

The overall distribution of the number of days until the next visit among all encounters were comparable before and during the pandemic and between in-person and telehealth visits. More than 60% of the next visits happened within 30 days. Proportionally more next visits happened on the 7^th^, 14^th^, 21^st^ and 28^th^ day from the initial visit (Fig. 1).

After adjusting for patient characteristics and month fixed effects, both telehealth and in-person visits had fewer number of days until the next visit during the pandemic relative to in-person visits before the pandemic (33.4 and 31.2 days respectively vs. 43.9 days; OR 0.76 [0.75,0.78] and 0.71 [0.70, 0.72]), but the two visit types were not significantly different from each other in number of days until the next visit. Similarly, the predicted probabilities of 3-, 7-, 14- and 30-day follow-up were higher during the pandemic period than before (OR 1.36–1.74). Comparing between telehealth and in-person visits during the pandemic, telehealth visits had comparable 3- and 7-day follow-up rates (9.2% [8.6%, 9.7%] vs. 9.3 [8.9%, 9.7%] for 3-day; 21.8% [21.0%, 22.5%] vs. 23.5% [22.0%, 24.1%] for 7-day) but lower 14- and 30-day follow-up rates than in-person visits during the pandemic (37.8% [36.9%, 38.6%] vs. 40.6% [40.1%, 41.3%] for 14-day; 62.4 (61.6, 63.2) vs. 65.1% [64.5%, 65.7%] for 30-day) (Table 3).

**Table 3:** Risk-Adjusted Time Duration (Number of Days) until the Next Visit and the Likelihood of Follow-up Visits

	Outpatient Encounters – Telehealth and In-Person (*N* = 134,375)		
	Baseline Pre-Pandemic (*N* = 89,357)	Study Period During Pandemic (*N* = 45,018)	
	In-person Visit (*N* = 89,357)	Telehealth Visit (*N* = 17,659)	In-person Visit (*N* = 27,359)
IRR/OR			
Number of Days until the next visit	ref	0.76 (0.75, 0.78)	0.71 (0.70, 0.72)
Probability, % (0–100)			
3-Day Follow-up	ref	1.36 (1.26, 1.47)	1.38 (1.30, 1.47)
7-Day Follow-up	ref	1.36 (1.29, 1.43)	1.51 (1.45, 1.57)
14-Day Follow-up	ref	1.39 (1.33, 1.45)	1.57 (1.52, 1.62)
30-Day Follow-up	ref	1.54 (1.48, 1.61)	1.74 (1.68, 1.79)
Predicted Outcomes			
Number of Days until the next visit	43.9 (43.6, 44.2)	33.4 (32.9, 33.9)	31.2 (30.8, 31.5)
Probability, % (0–100)			
3-Day Follow-up	6.9 (6.7, 7.1)	9.2 (8.6, 9.7)	9.3 (8.9, 9.7)
7-Day Follow-up	17.0 (16.7, 17.2)	21.8 (21.0, 22.5)	23.5 (22.0, 24.1)
14-Day Follow-up	30.5 (30.1, 30.8)	37.8 (36.9, 38.6)	40.6 (40.1, 41.3)
30-Day Follow-up	52.0 (51.7, 52.4)	62.4 (61.6, 63.2)	65.1 (64.5, 65.7)

*IRR* Incidence rate ratios, *OR* Odds ratios. 95% confidence interval in parentheses

Entries in the top panel were the estimated IRR (or OR) from regressions of number of days until the next visits (or whether a follow-up visit happened within 3, 7, 14, or 30 days) on the study period and the visit type (telehealth or in-person), with the baseline period and in-person visits as the default group. Entries in the bottom panel were the predicted number of days until the next visit and the predicted probabilities of 3-, 7-, 14- and 30-day follow-up from the regressions. All analysis were adjusted for patient characteristics and month fixed effects. Patient characteristics included age category (65–74 yrs., 75–85 yrs. or 86 + yrs.), sex, race/ethnicity (non-Hispanic White or Others), Medicaid coverage (0/1), disability entitlement (0/1), rural/urban residence (urban, suburban, large town, or small town/isolated rural), Hierarchical Condition Category (HCC) score and having 3 or more chronic conditions (0/1)

## Discussion

Using a longitudinal sample of Medicare beneficiaries who were 65 years or older and were served by an Accountable Care Organization, this study leveraged the COVID-19 pandemic as a natural experiment to examine the association of unplanned events, care costs and follow-up visit patterns with visit modality (telehealth vs. in-person) in primary care settings. This study first assessed the occurrence of non-COVID related unplanned events and the health care costs among patients with various types of outpatient encounters (no visit, telehealth only, in-person only and both types) during the pandemic relative to the pre-pandemic period. The study also estimated the time and likelihood of each type of encounter – telehealth or in-person visit that led to the next (follow-up) visit. Results suggested that patients used either telehealth or in-person services or both depending on their medical and personal accessibility and mainly treated telehealth as a substitute to in-person services rather than a replacement altogether. Telehealth was comparable to in-person services in primary care settings as it did not lead to more or sooner follow-up visits when compared to in-person encounters.

Our study contributes to the literature on the utilization and outcomes of telehealth in a few unique ways. First, to assess telehealth as a substitute to in-person services, we categorized patients into four mutually exclusive groups based on their outpatient care utilization during the pandemic period when telehealth first became available to the study sample. The categorization of patients into groups with no visits, telehealth only, in-person only and both types allowed us to compare telehealth only and in-person only groups directly against each other as well as jointly compare both with the group that used both types of services. Previous studies in the field which compared telehealth users versus non-users (or heavy users vs. lighter users) did not isolate the use of in-person services and hence, limited the capacity to identify expected variations in care outcomes, events, and costs across patient groups [24–27].

Second, the distribution of the four patient categories and the baseline information from the longitudinal feature of the study sample provided initial evidence that telehealth was treated as a substitute to in-person services. First off, the four patient categories took 18%, 18%, 30% and 34% of the sample respectively, with the telehealth only and in-person only groups jointly representing almost half of the study sample. Significant shares of patients with either type of service implied that many patients chose one type over the other. In addition, by tracing the patients back through the twelve month baseline period before the pandemic, results showed that patients with either type of visit, telehealth only or in-person only, were comparable in health conditions and care utilization during the baseline, and hence, the choice between telehealth and in-person services during the pandemic were mainly due to new health needs or existing social determinants such as barriers or facilitators (e.g., gender, age, rurality, technology, etc.). Lastly, patients who used both types of services during the pandemic had worse baseline health outcomes than the other three patient categories, which suggested that patients with different medical needs used telehealth differently. When telehealth is available, those healthier patients will use it to replace in-person visits altogether, and those less healthy (or more medically ill) ones will use it to replace some in-person services when necessary.

Third, to assess the associated occurrence of unplanned events with telehealth versus in-person service modality, this study investigated the patient-month level occurrence of non-COVID related unplanned events (i.e., ED visits and hospitalization) and health care costs across patient categories, as well as the encounter-level follow-up rates between the two encounter types. Both sets of results implied the potential of telehealth as a substitute to in-person services. Results from the patient-month data suggested that telehealth only and in-person only groups were comparable to each other in baseline events and care costs, and both significantly reduced unplanned events and care costs during the pandemic relative to their baseline levels. During the first phase of the COVID-19 pandemic it was expected that all visits to hospitals would be reduced to essential visits only. However, the telehealth only group had a larger reduction than the in-person group, and as a result, the two groups became significantly different in event occurrence and cost during the pandemic. While one cannot say telehealth is the cause of the improved outcomes [24, 25], we can still infer that when telehealth became available, patients (and providers) would have the ability to self-select the appropriate service type and save costs accordingly [3, 4]. Further, results from the encounter-level data suggested that compared to encounters before the pandemic, encounters initiated during the pandemic had fewer number of days until the next encounter and higher (3-, 7-, 14-, 30-day) follow-up rates, but telehealth encounters were not significantly different from in-person encounters for these metrics during the pandemic [26, 27].

### Limitations

This study has a few limitations. First, the study sample came from a single care system and could not represent the general aging population nor Medicare beneficiaries from managed care plans. However, focusing on one care system allowed us to leverage richer information from both electronic health records and claims data for analysis [49, 50]. Second, findings from the study suggested that telehealth was used as a substitute for in-person services and did not lead to sooner or more frequent services in the future. But as with many other observational studies, we cannot establish causal relationship between telehealth and care outcomes, such as unplanned events and follow-up visits. Some study designs to construct comparison groups or with randomized interventions would help. Third, for the analysis on the time duration until the next visit, we can only group the outpatient encounters by beneficiary and encounter date and cannot guarantee that any two consecutive encounters were for the same healthcare concerns. Dose response relationship across follow-ups beyond the immediate next visit was not considered either. Similarly, the data did not include information on the waiting time from requesting an appointment to being seen for telehealth or in-person visits, nor were data available to discern whether an ED visit or hospitalization was just a one-off event or due to a patient’s bypass of primary care. Using additional information on primary diagnosis and reason for the visit will help refine the results. Lastly, results from this study were based on the actual utilization data. Patients who faced barriers to access telehealth, such as technology or health literacy, had to stay with in-person services or no service at all during the pandemic period. As such, results from the study were conditional on telehealth availability and accessibility. More system and community efforts are expected to ensure equitable access to telehealth as an alternative to traditional in-person services in the long run.

## Conclusions

Using a sample of the US Medicare beneficiaries from one care system, this study found that patients and providers treated telehealth and in-person visits as substitutes and used either type depending on the medical needs, service availability and accessibility. For healthier patients without complicated medical needs, telehealth can substitute for in-person services altogether. For less healthy patients who need more outpatient visits or have more unplanned events, telehealth can substitute for in-person care for pre-diagnosis, monitoring, and/or follow-up. Further, telehealth did not lead to sooner or more follow-up visits than in-person services. For all patients and the healthcare system, telehealth works as an effective substitute to in-person services and has the potential to improve timely care access and reduce health costs. Evidence obtained from the US healthcare market on the potential benefits of telehealth also yield similar implications to other countries in the world during the pandemic and beyond.

## Supplementary Information

**Additional file 1: Supplementary Table 1.** Unadjusted Occurrence of Unplanned Events and Medicare Payments per Month.

## Data Availability

The data that support the findings of this study are available from the Center for Medicare and Medicaid Services (CMS), but restrictions apply to the availability of these data, which were used under license for the current study, and so are not publicly available. Data are however available from the authors upon reasonable request and with permission of CMS.

## Abbreviations

ED: Emergency Department
ACO: Accountable Care Organization
CMS: The Centers for Medicare & Medicaid Services
HCC: Hierarchical Condition Categories
IRR: Incidence Rate Ratios
OR: Odds Ratios
SD: Standard Deviation
CI: Confidence Interval
