# Supplementary material for: Use telehealth as needed: telehealth substitutes in-person primary care and associates with the changes in unplanned events and follow-up visits
Source: BMC Health Serv Res. 2023 May 3;23:426. doi: 10.1186/s12913-023-09445-0 (PMC10154749; doi:10.1186/s12913-023-09445-0)
Supplement: Supplementary file 1 — Additional file 1: Supplementary Table 1. Unadjusted Occurrence of Unplanned Events and Medicare Payments per Month. [file 12913_2023_9445_MOESM1_ESM.docx]

**Supplementary Table 1**: Unadjusted Occurrence of Unplanned Events and Medicare Payments per Month

|  | Patient-Month Records (N = 338,872) | | | |
| --- | --- | --- | --- | --- |
|  | No Visits  (N = 61,548) | Telehealth Only  (N = 61,235) | In-person Only  (N = 101,695) | Both Types  (N = 114,394) |
| Prob (ED=1) per month (%) | | | | |
| Baseline | 1.90 (1.76, 2.04) | 2.20 (2.05, 2.35) | 2.16 (2.05, 2.28) | 4.04 (3.89, 4.19) |
| Study Period | 0.65 (0.56, 0.75) | 1.18 (1.04, 1.30) | 1.80 (1.67, 1.92) | 3.92 (3.74, 4.09) |
| Difference | -1.25 (-1.48, -1.01) *** | -1.02 (-1.31, -0.75) *** | -0.36 (-0.61, -0.13) *** | -0.12 (-0.45, 0.2) |
|  |  |  |  |  |
| # of ED per 1,000 patients per month | | | | |
| Baseline | 20.9 (19.3, 22.6) | 24.0 (22.3, 25.8) | 23.7 (22.4, 25.1) | 45.7 (43.8, 47.5) |
| Study Period | 6.9 (5.8, 7.9) | 12.3 (10.9, 13.7) | 19.1 (17.8, 20.5) | 45.0 (42.8, 47.1) |
| Difference | -14 (-16.8, -11.4) *** | -11.7 (-14.9, -8.6) *** | -4.6 (-7.3, -1.9) *** | -0.7 (-4.7, 3.3) |
|  |  |  |  |  |
| Prob (Hospitalization=1) per month (%) | | | | |
| Baseline | 0.89 (0.79, 0.98) | 1.13 (1.02, 1.24) | 0.95 (0.88, 1.03) | 1.94 (1.83, 2.04) |
| Study Period | 0.35 (0.28, 0.42) | 0.66 (0.56, 0.76) | 0.84 (0.75, 0.93) | 2.30 (2.16, 2.43) |
| Difference | -0.54 (-0.7, -0.37) *** | -0.47 (-0.68, -0.26) *** | -0.11 (-0.28, 0.05) | 0.36 (0.12, 0.6) ** |
|  |  |  |  |  |
| # of Hospitalization per 1,000 patients per month | | |  |  |
| Baseline | 9.3 (8.3, 10.4) | 11.8 (10.6, 13.0) | 9.9 (9.1, 10.8) | 20.5 (19.3, 21.6) |
| Study Period | 3.8 (3.0, 4.6) | 6.9 (5.8, 7.9) | 8.9 (8.0, 9.8) | 24.5 (23.0, 26.0) |
| Difference | -5.5 (-7.4, -3.7) *** | -4.9 (-7.2, -2.7) *** | -1.0 (-2.8, 0.7) | 4.0 (1.4, 6.7) *** |
|  |  |  |  |  |
| $ Unplanned payment per patient over the study period | | | | |
| Baseline | 663 (643, 683) | 1176 (1141, 1212) | 1036 (1012, 1060) | 2899 (2835, 2963) |
| Study Period | 298 (288, 307) | 726 (701, 750) | 840 (817, 862) | 3264 (3180, 3347) |
| Difference | -365 (-395, -336) *** | -450 (-511, -391) *** | -196 (-243, -150) *** | 365 (217, 512) *** |

Note: 95% confidence interval in parentheses. *** 99%, ** 95%, * 90% significant level.

Entries are predicted occurrence of unplanned events (Emergency Department (ED) visits and hospitalization) and Medicare payment after risk adjustment of patient characteristics and month fixed effects. Occurrence of ED visits (or hospitalization) were measured by the average probability of having at least 1 ED visits (or hospitalizations) per patient per month and the average number of ED visits (or hospitalizations) per 1,000 patients per month. Medicare payment was measured by the average dollar amount per patient per month. Patient characteristics included age category (65-74 yrs., 75-85 yrs. or 86+ yrs.), sex, race/ethnicity (non-Hispanic White or Others), Medicaid coverage (0/1), disability entitlement (0/1), rural/urban residence (urban, suburban, large town, or small town/isolated rural), Hierarchical Condition Category (HCC) score and having 3 or more chronic conditions (0/1)
